# Integrating network pharmacology prediction and experimental investigation to verify ginkgetin anti-invasion and metastasis of human lung adenocarcinoma cells *via* the Akt/GSK-3β/Snail and Wnt/β-catenin pathway

**DOI:** 10.3389/fphar.2023.1135601

**Published:** 2023-03-02

**Authors:** Kaile Liu, Xiaojie Fu, Zhongqi Wang, Lian Yang, Jia Yang, Haibin Deng

**Affiliations:** Department of Oncology, Longhua Hospital, Shanghai University of Traditional Chinese Medicine, Shanghai, China

**Keywords:** Ginkgetin, lung adenocarcinoma cells, AKT/GSK-3β/Snail signaling pathway, Wnt/β-catenin signaling pathway, network pharmacology

## Abstract

**Introduction:** Lung cancer, one of the most frequent malignancies, has a high death rate and an increased number of new cases globally. Ginkgo biloba has been used for many years in the treatment of lung cancer. Ginkgetin is the key active ingredient extracted from Ginkgo biloba. However, the mechanism by which ginkgetin inhibits the invasive metastasis of lung cancer is unclear.

**Methods:** We used a network pharmacology approach to obtain the molecular mechanism by which ginkgetin inhibits lung cancer metastasis. Then we analyzed potential target proteins between ginkgetin and lung cancer. Finally, we validated with molecular docking and experimental validation.

**Results:** By analyzing the intersecting genes of lung cancer and ginkgetin, there were 79 intersecting genes, which were mainly involved in the positive regulation of cell migration, with the cancer pathway being one of the most enriched pathways. The results of *in vitro* experiments showed that GK had a large inhibitory effect on cell invasion and metastasis of A549 and H1299. *In vivo* animals GK had a great inhibitory effect on metastasis of LLC.

**Conclusion:** This study identified the potential related GK molecular targets and signaling pathways in treating human lung cancer using network pharmacological approaches. Experiments confirmed that GK inhibits the Akt/GSK-3β/Snail and Wnt/β-catenin cascade initiation in A549, H1299 and LLC cells, preventing metastasis. This study’s results align with the hypotheses derived from the network pharmacology analysis.

## 1 Introduction

Cancer is a public health problem that affects people worldwide. Experts predict a 50% increase in new cancer cases globally over the next two decades (Mao et al., 2022). Lung cancer, one of the most frequent malignancies, has a high death rate and an increased number of new cases globally. In the next 20 years, the number of new cancer cases globally will be projected to increase by around 50%. Lung cancer is divided into small cell lung cancer (SCLC) and non-small cell lung cancer (NSCLC). Different types and stages of lung cancer require different treatments (Song et al., 2022). It is believed that there are now 654,620 men and women living in the United States with a history of lung cancer, with an additional 236,740 cases expected in 2022. SCLC and NSCLC account for 14% and 82% of all successfully treated cases, respectively. Approximately 3% of these cases have undefined histology (Miller et al., 2022). The development of resistance to chemotherapeutic medications and the occurrence of adverse responses to these treatments have become major challenges in modern oncology (Efferth et al., 2008). Therefore, creating cancer treatments that work more quickly and effectively is important.

Ginkgetin (Ginkgo biloba L. [Ginkgoaceae], World Checklist, Ginkgetin, GK), C_32_H_22_O_10_, firstly isolated from *Ginkgo biloba* extract in 1929, is a natural biflavone with no toxicity (Gontijo et al., 2017). GK regulates its antitumor effectiveness through different approaches and processes, including cell cycle arrest (Liu et al., 2022a), inhibiting proliferation (Cheng et al., 2019), inducing apoptosis (Hu et al., 2019), triggering autophagy (Lou et al., 2017), and preventing angiogenesis (Hu et al., 2019). Moreover, GK has anticancer characteristics against hepatocellular carcinoma (Liu et al., 2022b), leukemia (Pan et al., 2017), and medulloblastoma (Ye et al., 2015), including colon (Hu et al., 2019), neck, prostate (Jeon et al., 2015), breast (Park et al., 2017), cervical (Cheng et al., 2019), ovarian (Su et al., 2000), and myeloma cancers (Liu et al., 2022a). However, the primary cause of this problem is that the regulatory mechanism of GK on lung cancer is unclear. Therefore, there is a critical need to investigate and research the GK-related molecular targets and signaling pathways in lung cancer. Identifying prospective targets and pathways to clarify the mechanism of GK on lung cancer is of utmost importance since cancer is a complicated process caused by several genes and risk exposure factors.

Through multi-objective, multi-channel, and multi-link analysis, network pharmacology in a web-based format shows the advanced pharmacological mechanisms of traditional Chinese medicine (TCM) (Li et al., 2015). In the present study, our team used the network pharmacology technique to access the molecular pathways behind GK’s ability to block lung cancer invasion and metastasis.

## 2 Materials and methods

### 2.1 Materials and reagents

GK was purchased from MCE co, Ltd. (Shanghai, China). Cell Signaling Technology provided the primary antibodies used in this study: anti-N-cadherin (#13116), anti-Vimentin (#5741), anti-Snail (#3879), anti-p-AKT (#4060), anti-AKT (#4691), anti-GSK-3β (#12456),anti-p-GSK-3β(#5558),anti-actin (#4970), and anti-rabbit IgG HRP (#7074) (Danvers, MA, United States). Anti-c-Myc (ab32072) purchased from Abcam (Cambridge, United Kingdom)). Gibco provided the trypsin-EDTA used in this study (California, United States). CWBio supplied the RIPA Lysis Buffer and the ECL Western blot kit (Taizhou, Jiangsu, China). Hyclone was consulted to acquire the minimum necessary medium (MEM) (Logan, UT, United States). The Beyotime Cell Counting Kit-8 was purchased (Shanghai, China).

### 2.2 Target prediction of GK

We used the PharmMapper (http://www.lilab-ecust.cn/pharmmapper) platform to predict GK targets, backed by a sizable in-house pharmacophore database extracted from all targets in TargetBank, DrugBank, BindingDB, and PDTD, providing storage for and access to >7,000 receptor-based pharmacophore models (covering information on 1,627 drug targets, 459 of which are human protein targets). The top N possible drug targets and the alignment pose for each molecule were generated after the user uploaded molecules (in Tripos Mol2 or MDL SDF format) with the best mapping posture to all targets in PharmTargetDB.

### 2.3 Determining the targets of GK in lung cancer

We obtained lung cancer-related targets from MalaCards. The MalaCards (https://www.malacards.org) is a database of diseases specific to humans from GeneCards, containing and organizing >20,000 disease entries from more than 80 data sources, including OMIM and orphanet. The database displays a “disease card” for each disease, with various information regarding the disease, including disease aliases, related diseases, therapeutic agents, anatomical background, and disease-related genes. Using the Uniprot (https://www.uniprot.org) database, we converted the names of drugs’ active components into gene symbols. Finally, we found shared targets between GK and lung cancer using a screening process. All procedures followed were according to the principles of the Declaration of Helsinki.

### 2.4 Enrichment analysis of common targets of GK and lung cancer

The Metascape (https://metascape.org) platform was used to conduct pathway enrichment analyses based on the Gene Ontology (GO) and Kyoto Encyclopedia of Genes and Genomes (KEGG) to identify the biological processes and signaling pathways associated with the targets shared by GK and lung cancer. The biomedical researchers’ community may evaluate gene/protein lists and make better data-driven decisions with the support of Metascape’s trustworthy, effective, and user-friendly tool collection.

### 2.5 Construction of GK, targets, and pathway network

The STRING database (https://string-db.org) is used to predict network interactions between proteins. The most frequently reported targets for GK and lung cancer were provided on this site to compile PPI network maps and information. Sharing targets and pathways led to establishing a GK network model using Cytoscape 3.9.0.

### 2.6 Molecular docking

We download the 3D structure of Ginkgo biflavone in SDF format from PubChem data, import the structure into ChemBio3D Ultra 14.0 for energy minimization, set the Minimum RMS Gradient to 0.001, and save the small molecule in mol2 format. Import the optimized small molecule into AutodockTools-1.5.6 for hydrogenation, charge calculation, charge assignment, and rotatable key setting, and then save it as “pdbqt” format. Download the protein structures of AKT1 (PDB ID: 1UNQ), CTNNB1 (PDB ID: 1JDH) and GSBK3β (PDB ID: 6V6L) from the PDB database; use Pymol 2.3.0 to remove protein crystalline water, original ligands, etc., and import the protein structures into AutoDocktools (v1.5.6) for hydrogenation, charge calculation, charge assignment, and assigning rotatable keys. The protein structure is imported into AutoDocktools (v1.5.6) for hydrogenation, charge calculation, charge assignment, atom type specification and saved in “pdbqt” format. Prediction of protein binding sites using POCASA 1.1 and docking using AutoDock Vina1.1.2.

### 2.7 Cell line and cell culture

Lung adenocarcinoma cells (A549, H1299 and LLC) were acquired from the American Type Culture Collection (Manassas, VA, United States) and grown at 37°C in humid air with 5% carbon dioxide (CO_2_) in Dulbecco’s Modified Eagle Medium supplemented with 10% fetal bovine serum (FBS), 100 U/mL penicillin, and 100 g/mL streptomycin.

### 2.8 Cell viability assay

To test GK’s cytotoxicity against A549 and H1299 cells, we used the CCK-8 assay to determine the viability of the 2 cells. These 2 cells were seeded onto 96-well plates at a density of 3,000 cells/well. Three plates were created under the same conditions as repeat experiments and incubated overnight at 37°C in humid air containing 5% CO_2_. The cells were treated for 72 h with GK at the gradient-varying concentrations indicated (0, 2.5, 5, and 10 µM). Subsequently, we added CCK-8 solution to each well and incubated the samples under the same conditions for 1 h. The cell growth vitality in each well was measured at 450 nm using a multi-mode reader (BioTek, VT, United States).

### 2.9 Wound healing assay

In order to explore the effect of GK on human lung adenocarcinoma cells (A549 and H1299) migration, we did the wound healing experiment, using a marker pen and a straightedge to draw horizontal lines evenly across the back of the 6-well plate, approximately every 0.6 cm, across the wells, with five lines across each well, adding approximately 5×10^5^ cells to the wells, and after the cells were spread across the 6-well plate, we pretreated the plate with 1 μg/ml mitomycin C for 1 h, and then performed the scratching experiment with a 200 μL gun tip with five lines perpendicular to it. The cells were then washed three times with phosphate-buffered saline to remove the scratched-down cells; the adherent cells were cultured in FBS-free, a free medium containing the indicated GK concentrations (0,2.5, 5, and 10 µM). The cells’ migration distance on the wound was observed at 0 and 24 h, measured using a microscope and photographed for documentation.

### 2.10 Transwell migration assays

To demonstrate further modifications of GK-induced migratory capability on human lung adenocarcinoma cells (A549 and H1299), we performed Transwell migration research using Transwell chambers (Corning, NY, United States). Cells were pretreated with 10 μg/mL of mitomycin C for 1 h in serum-free medium, the upper chamber was injected with 2 × 10^4^ cells and kept at 37°C.Dimethyl sulfoxide or GK was applied to the cells at the appropriate concentrations (0, 2.5, 5, and 10 µM). To induce cell migration to the lower chamber, media containing 20% FBS was added to the bottom of the cell chamber. Cells were fixed with paraformaldehyde and stained with 0.5% crystal violet after 24 h. Lastly, a light microscope (×100 magnification, Olympus, Tokyo, Japan) was used to view, photograph, and count the cells.

### 2.11 Western blot analysis

Total cellular proteins treated with various GK doses were extracted using RIPA lysis buffer and subjected to protein electrophoresis using SDS-PAGE, followed by protein transfer to PVDF membranes (Merck Millipore Ltd., Tulla Green, Ireland). TBST solution with 5% skimmed milk was used to block the PVDF membranes. The PVDF membranes were treated with a primary antibody (1:1,000) overnight at 4°C, followed by three washes with TBST and incubation with an HRP-conjugated secondary antibody (1:5,000) for 1 h. Finally, the PVDF membranes were photographed using a Tanon 5200 imager (Shanghai, China). To discover Immune complexes, utilize an ECL kit (Share Bio, Shanghai, China).

### 2.12 Animal experiments

Male C57BL/6 wild-type mice (7 weeks old, weighing 18–20 g, Shanghai. China) were purchased from Shanghai Jihui Laboratory Animal Care Co.,Ltd. Mice were housed and maintained in an animal facility that adhered to specific pathogen-free (SPF) standards. All animal experiments were performed in accordance with the Guide for Ethical Review of Laboratory Animal Welfare (GB/T 35892). All animal experiments were conducted in accordance with the “Guide for Ethical Review of Laboratory Animal Welfare (GB/T 35892-2018)”. All experimental procedures were approved by the Animal Care and Use Committee of Shanghai University of Traditional Chinese Medicine. All procedures were approved by the Animal Care and Use Committee of Shanghai University of Traditional Chinese Medicine (PZSHUTCM220919016). 1.5 × 10^5^ LLC cells were injected through the tail vein, and C57 mice were given the next day intraperitoneally with GK (15 mg/kg or 30 mg/kg) once daily or cisplatin (2 mg/kg). Intraperitoneal injection was given once every 2 days. Mice were euthanized after 2 weeks, and lungs were taken for follow-up investigation.

### 2.13 H&E staining

Lung tissue from mice was immersed and fixed in 10% formalin for 24 h and embedded in paraffin embedded and cut into 4 µm thick sections. h&E staining was performed in 4 µm paraffin sections were stained using standard H&E staining protocols. Final. Slides were sealed with neutral adhesive; tissue morphology was then observed and photographed under light microscopy.

### 2.14 Statistical analysis

The statistical findings are reported as the mean ± standard deviation. The Student’s *t*-test was performed to determine whether or not there was a significant difference between the two groups. We determined the statistical significance of group differences using GraphPad Prism8 software (GraphPad Software, Inc., San Diego, CA, United States). *p*-values <0.05 were considered statistically significant.

## 3 Results

### 3.1 Drug-target network analysis

**FIGURE 1:**
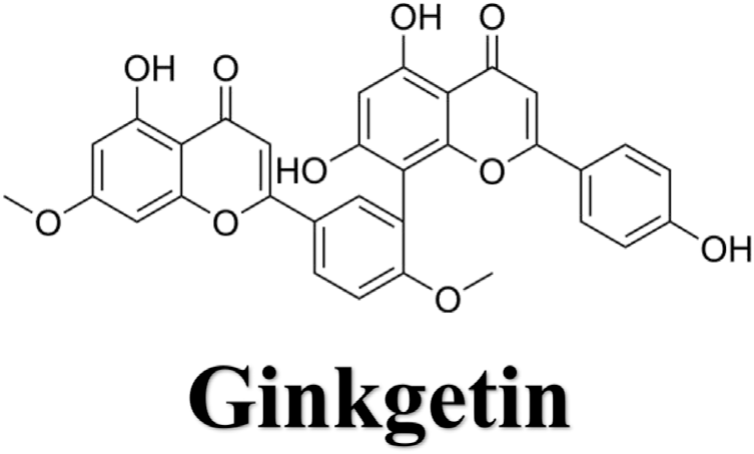
Molecular structure of ginkgetin (GK).

**FIGURE 2:**
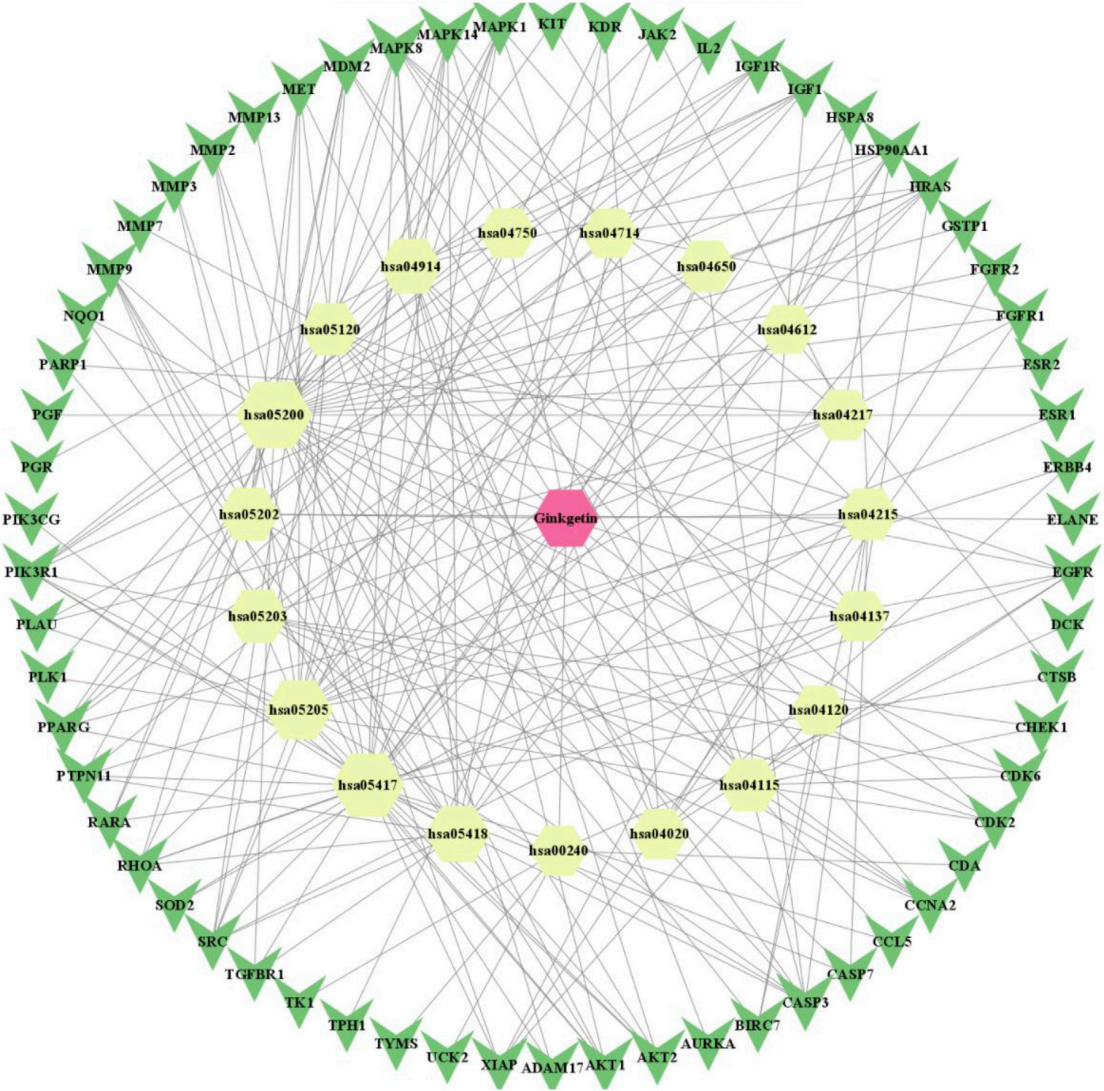
“Drug-compound-target (including PPI)-pathway” network diagram of Ginkgetin for lung cancer (red positive hexagon is Ginkgetin; yellow positive hexagon is the enriched pathway; green triangle is the intersecting gene).

We utilized network pharmacology to identify the GK targets that decreased lung adenocarcinima cells (A549, H1299, and LLC) activity and migration. Figure 1 showed the molecular structure of ginkgetin. We constructed a network of GK and their targets using the PharmMapper server. We obtained 297 targets from the PharmMapper server (Figure 2).

### 3.2 Common targets of the GK and lung cancer

**FIGURE 3:**
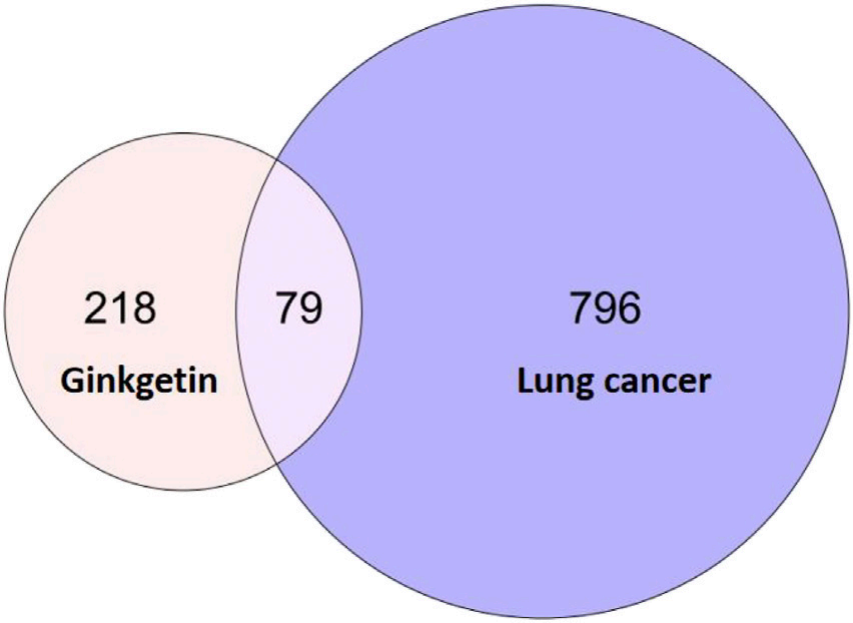
Venn diagram of a common target for lung cancer and GK.

On the PharmMapper platform, we acquired 297 GK-related targets. Similarly, we obtained 875 target genes related to lung cancer using the MalaCards database. We obtained 79 similar targets by crossing the active ingredient targets with disease-related ones (Figure 3).

### 3.3 Analysis of the KEGG pathway of GK and the enrichment of GO

The *p*-value was set to 0.01, and the lung cancer and GK’s shared targets were submitted to the Metascape platform. For the enrichment study of GO biological processes, cellular components, and molecular functions, 1,051 GO biological processes, 64 GO cellular components, 91 GO molecular functions, and 154 KEGG pathways were employed.

### 3.4 KEGG pathway analysis

**FIGURE 4:**
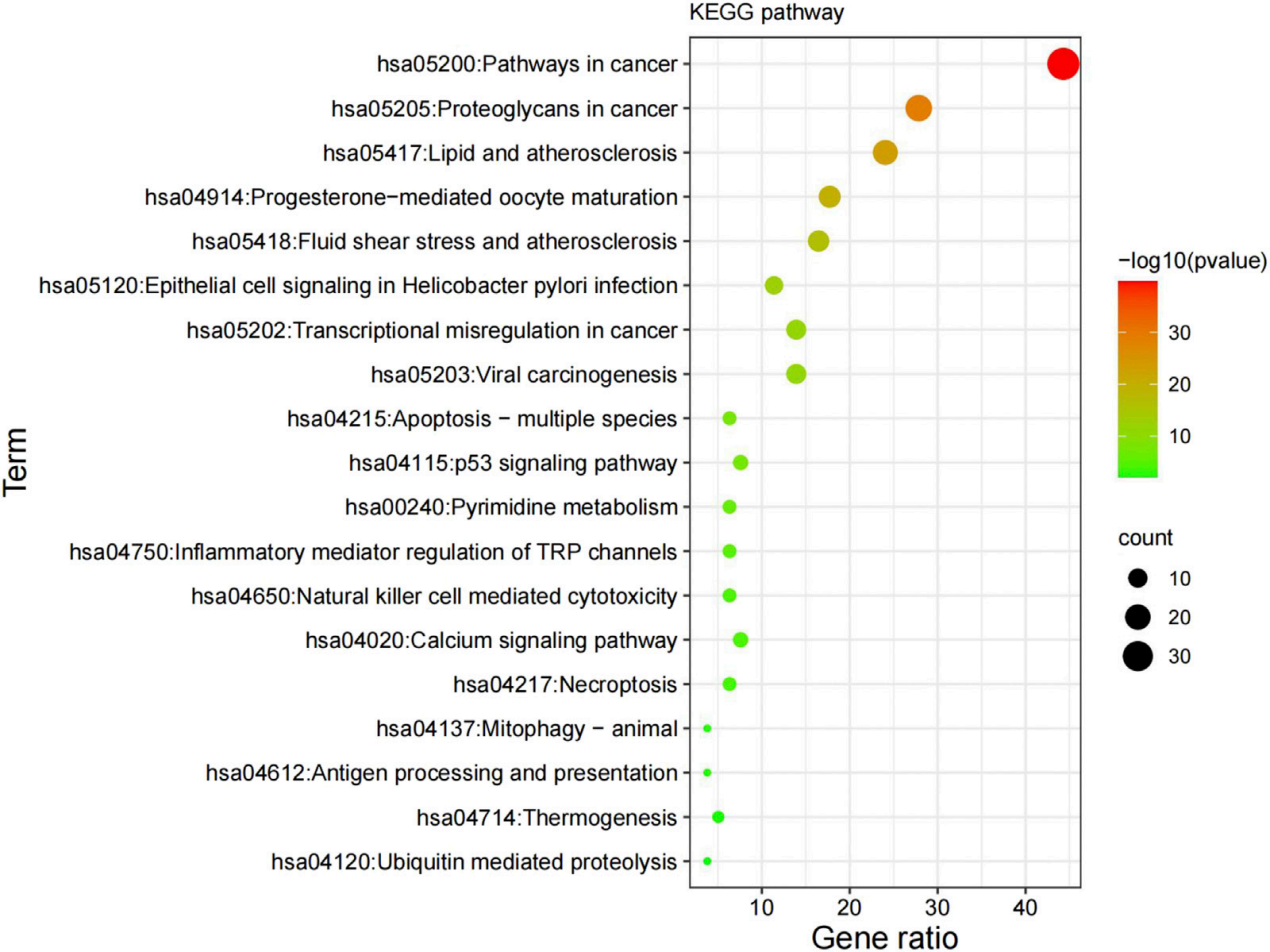
Results of the KEGG enrichment analysis. KEGG, Kyoto Encyclopedia of Genes and Genomes.

The KEGG pathway analysis uncovered 154 highly-enriched pathways, including cancer pathways, proteoglycans in cancer, transcriptional dysregulation in cancer, viral oncogenesis, apoptosis-polymorphic, and p53 signaling pathways. All KEGG routes were chosen for mapping and analysis. Figure 4 shows the top 20 most enriched routes. Potential GK treatment targets in lung cancer were primarily concentrated along the cancer process (hsa05200) (Figure 4).

### 3.5 GO analysis

**FIGURE 5:**
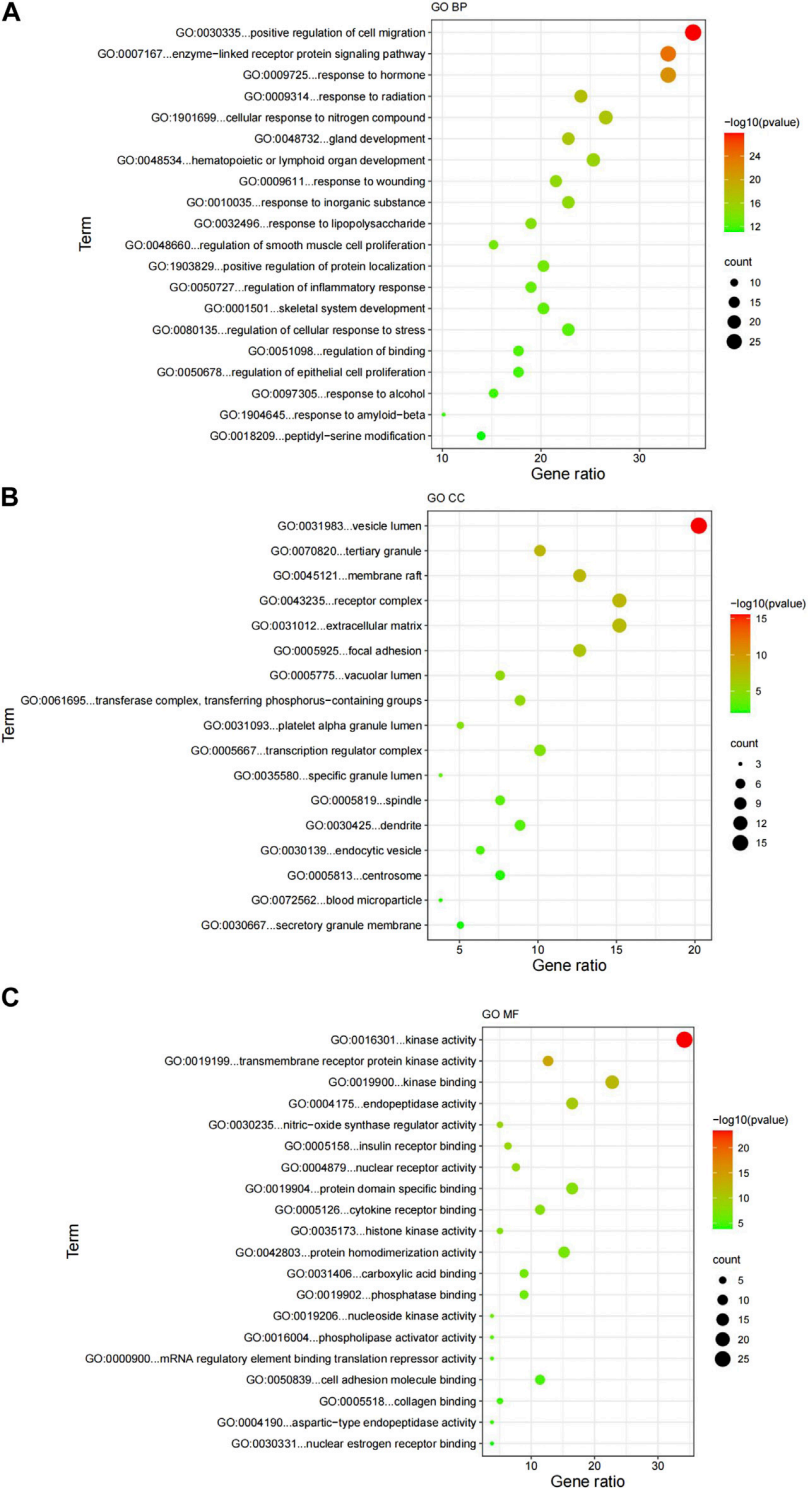
Results of the GO enrichment analysis. **(A)** GO biological processes; **(B)** GO cellular components; **(C)** GO molecular functions. GO, Gene Ontology; BP, biological processes; CC, cellular components; MF, molecular functions.

Figure 5 showed the top 20 enriched GO keywords in each sub-ontology. Highly enriched GO keywords included cell migration, signaling pathways, epithelial cell proliferation, receptors, kinases, mRNA translation inhibitors, and cell adhesion molecules. Further analysis of the top 20 GO biological processes, cellular components and molecular function enrichment revealed that the potential GK targets for lung cancer treatment primarily involve investigating its mechanism of action on lung cancer cell migration and invasion. Using the STRING database, we developed a protein interaction network of crossover genes, submitted the highest confidence protein interaction scores, and evaluated the key target, AKT.

### 3.6 PPI interaction network of common targets in GK and lung cancer

**FIGURE 6:**
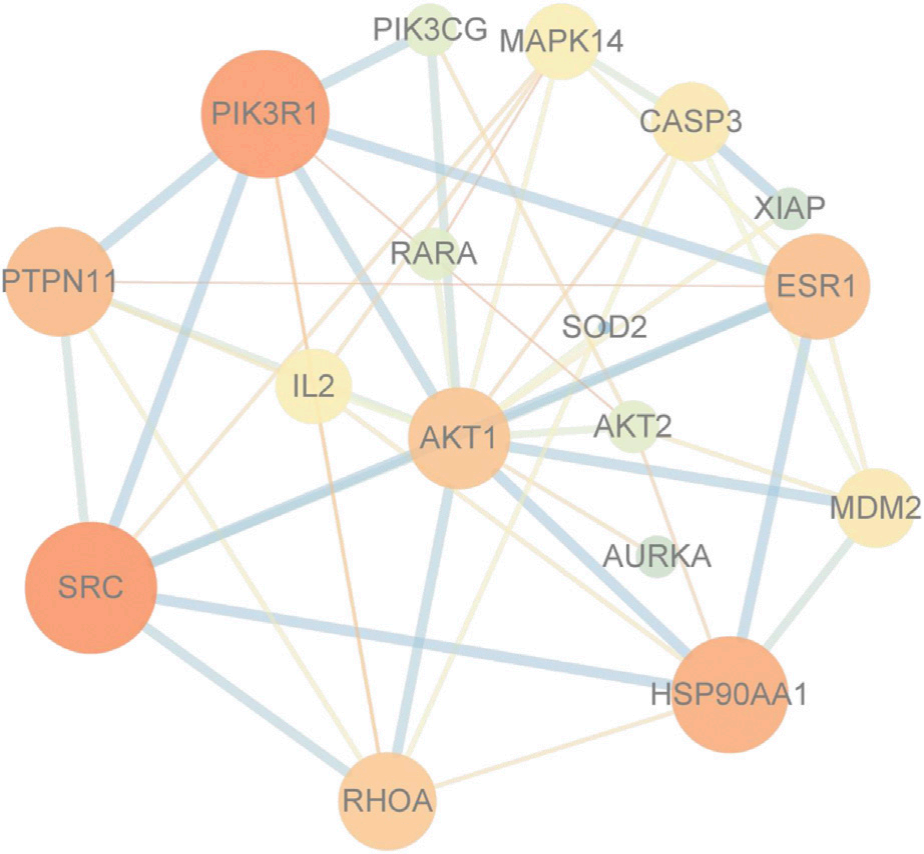
Protein-protein interaction network diagram. Core target AKT.

The PPI interaction network information was gathered by uploading targets to the STRING database and combining the data in Cytoscape 3.90. There are 99 vertices (drug: 1 node; target: 79 nodes; pathway: 19 nodes) (Figure 6).

### 3.7 Molecular docking results

**FIGURE 7:**
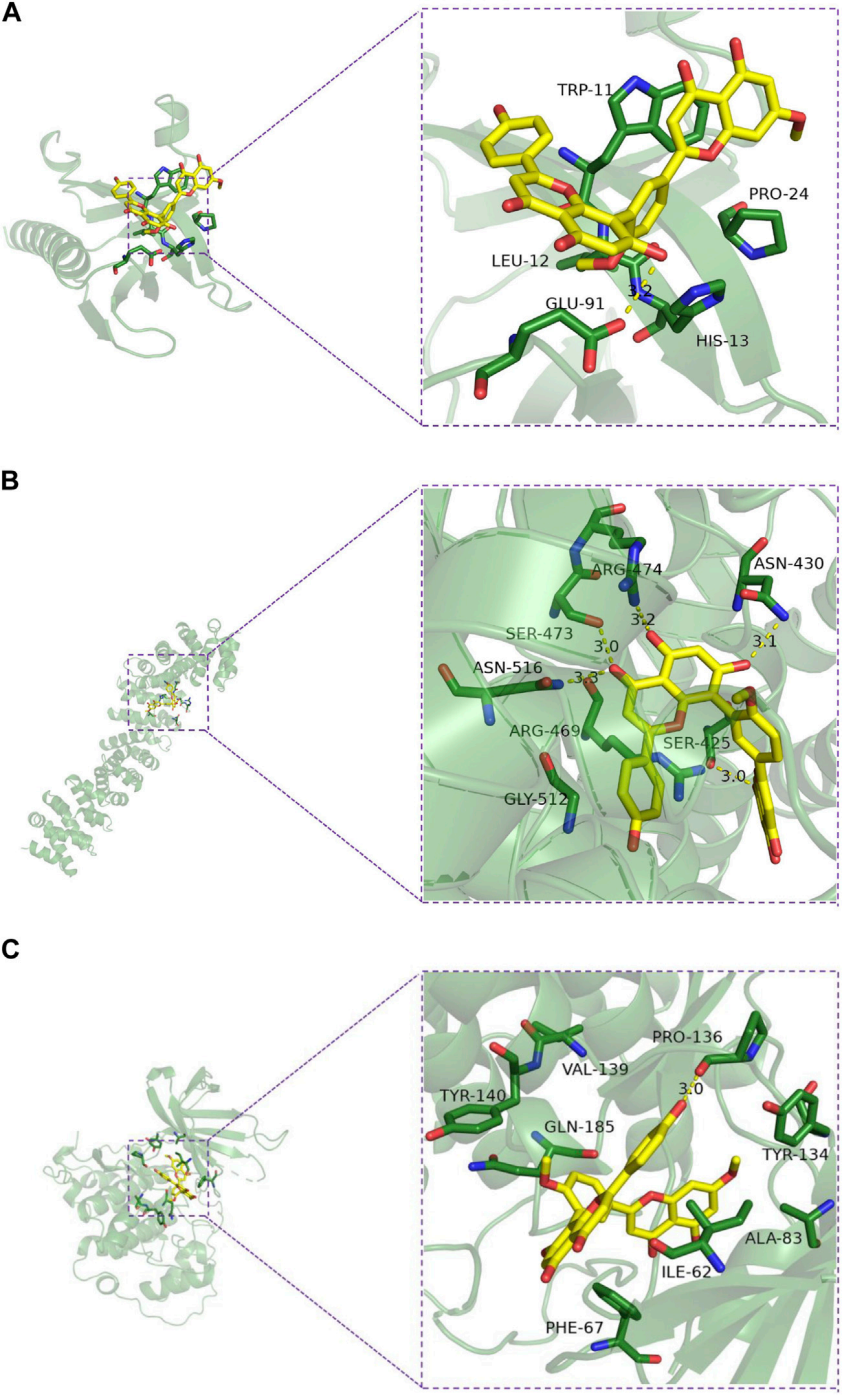
Molecular docking model diagram of the key substances-core targets. **(A)** Ginkgetin-Akt1; **(B)** Ginkgetin-β-catenin; **(C)** Ginkgetin-GSK-3β.

The interaction pattern analysis of the docking results using PyMOL2.3.0 showed that the binding energy of ginkgo biflavone with AKT1 was −7.6 kcal/mol, which proved to have a good binding effect. Ginkgo biflavone interacted with AKT1 mainly through the formation of hydrogen bonds as well as hydrophobic forces, forming hydrogen bonds with GLU-91 with a length of 3.2 Å; and hydrophobic interactions with HIS-13, PRO-24, TRP-11 and LEU-12 (Figure 7A). The binding energy of ginkgo biflavone with CTNNB1 was −8.8 kcal/mol, which proved to have a good binding effect. Ginkgo biflavones interacted with CTNNB1 mainly through the formation of hydrogen bonds as well as hydrophobic forces with SER-425, ASN-430, ARG-474, SER-473, ASN-516, ARG-469 with hydrogen bond lengths of 3.0Å, 3.1Å, 3.2Å, 3.0Å, 3.3Å, respectively; with ARG-469, GLY-512 have hydrophobic interaction (Figure 7B). The binding energy of ginkgo biflavone with GSK3β was −8.9 kcal/mol, which proved to have a good binding effect. Ginkgo biflavone interacted with GSK3β mainly through the formation of hydrogen bonds as well as hydrophobic forces, forming hydrogen bonds with PRO-136 with a length of 3.0 Å; and hydrophobic interactions with TYR-134, ALA-83, ILE-62, PHE-67, GLN-185, TYR-140, VAL-139 (Figure 7C).

### 3.8 GK inhibited A549 and H1299 cells viability

**FIGURE 8:**
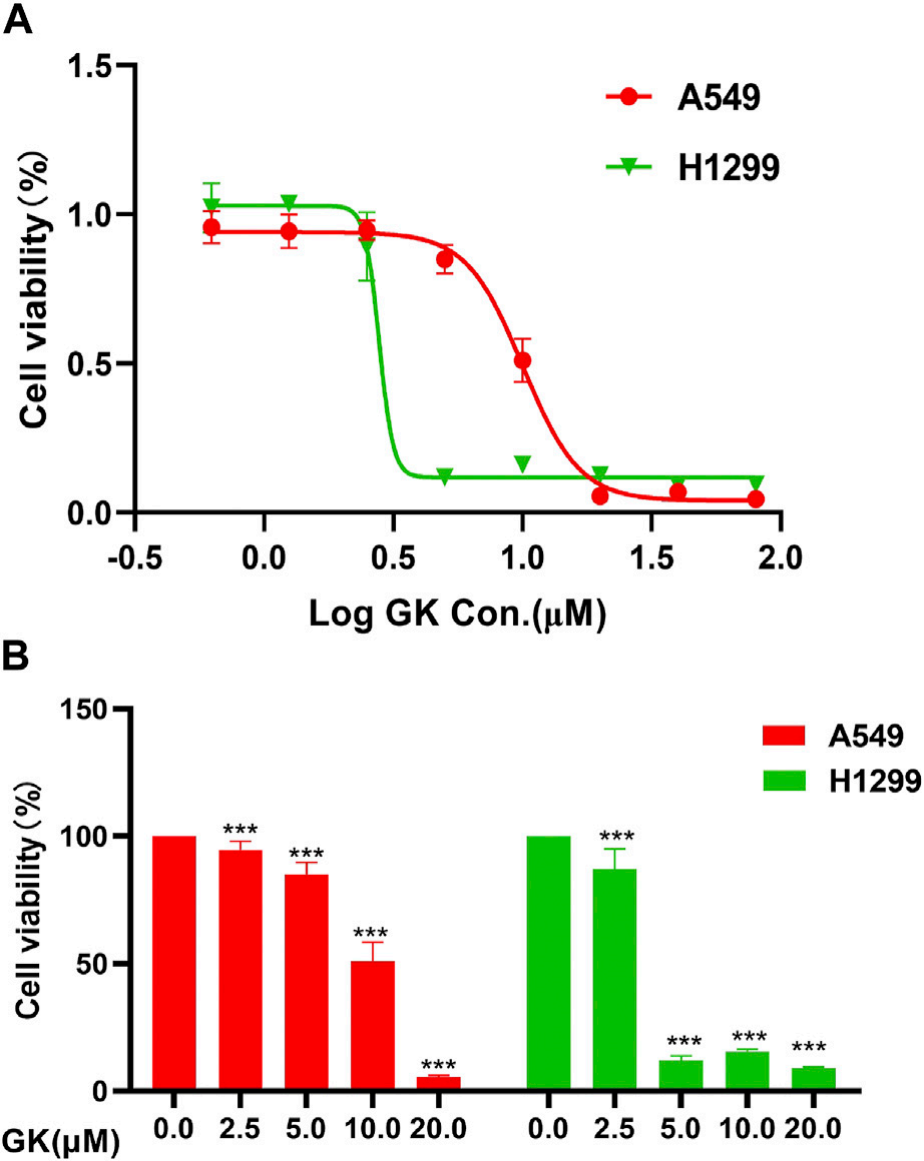
GK inhibits the proliferation of A549 and H1299 cells. **(A)** Growth curves obtained in proliferation (CCK-8) assays in the indicated A549 and H1299 cells. **(B)** Statistical analysis of the CCK-8 of the expressed A549 and H1299 cells.

We carried out the Cell Counting Kit-8 assay to verify the anti-proliferative effect of GK on A549 and H1299 cells. We treated these two types of human lung adenocarcinoma cells with cultures containing different GK concentrations for 72 h. The results demonstrated that GK significantly inhibited the proliferative activity of A549 and H1299 cells in a concentration-dependent manner. According to CCK-8 test data, GK’s IC_50_ on A549 and H1299 cells for 72 h was determined to be 10.05 μM and 2.789 μM (Figure 8).

### 3.9 GK hindered migration of A549 and H1299 cells

**FIGURE 9:**
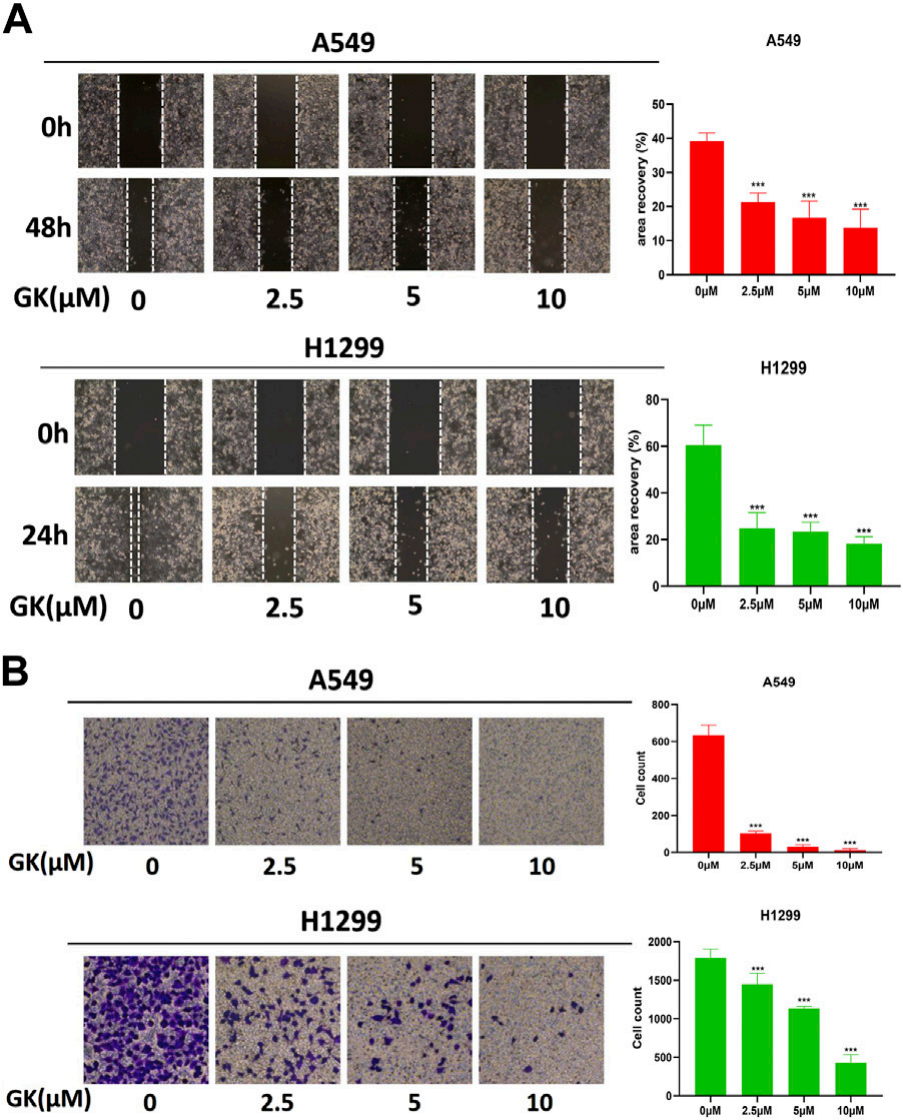
GK represses metastasis of A549 and H1299 cells *in vitro*. **(A)** Migration of A549 and H1299 cells, treated as indicated, was analyzed in wound-healing assays. **(B)** Transwell assay was used to assess the effect of GK on cell migration (magnification ×100).

The wound healing experiment revealed that the cell wound width was considerably lesser after 24 h or 48 h than at 0 h in the control group. In contrast, there was no significant difference between that at 0 and 24 or 48 h in the GK-treated group’s wound breadth (Figure 9A). In addition, we performed the transwell assay, and the results indicated that GK effectively attenuates migratory capacity of A549 and H1299 cells. GK markedly and dose-dependently inhibited the migration of A549 and H1299 cells (Figure 9B).

### 3.10 GK reduced the expression of mesenchymal cell markers in A549 and H1299 cells

**FIGURE 10:**
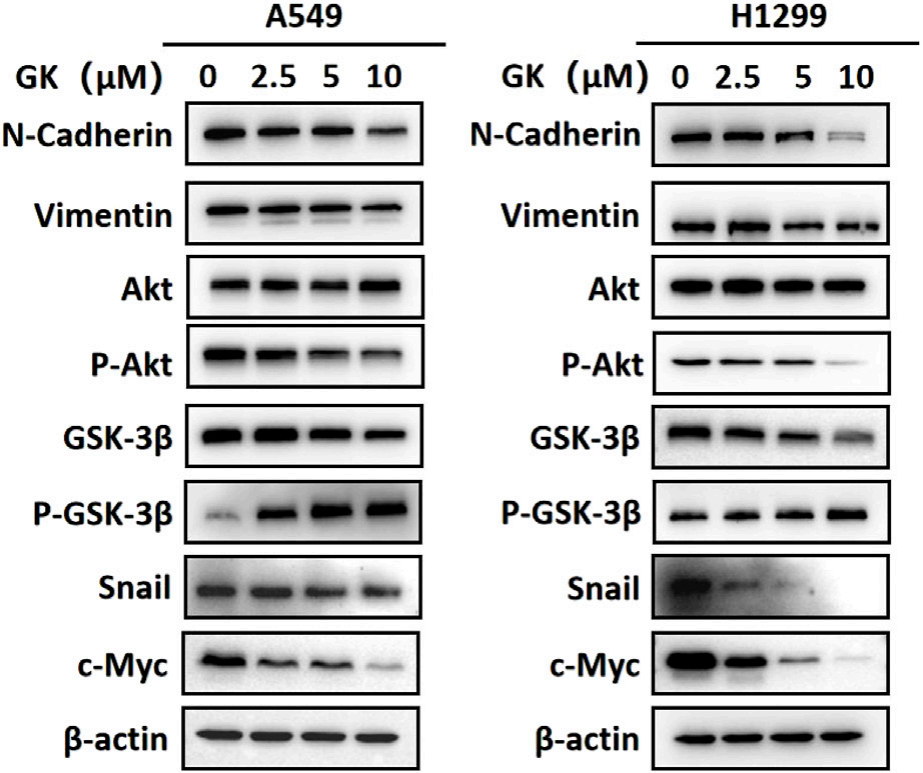
GK inhibits EMT in A549 and H1299 cells by modulating the AKT/GSK-3β/Snail pathway and Wnt/β-catenin pathway. Western blotting analysis of protein expression in the indicated A549 and H1299 cells.

After Western blotting analysis, we found that GK markedly inhibited N-cadherin and vimentin expression at the protein level in A549 and H1299 cells (Figure 10).

### 3.11 GK inhibited epithelial-to-mesenchymal transition (EMT) by inhibiting Akt/GSK-3β/Snail and Wnt/β-catenin activation in A549 and H1299 cells

EMT is the process by which epithelial cells undergo a phenotypic switch to that of a mesenchymal cell type. As a result of EMT, epithelial cells lose their polarity and attachment to the basement membrane but gain the ability to migrate, invade, resist apoptosis, and degrade the extracellular matrix. Western blotting analysis showed that the expression levels of p-Akt (ser473) and snail protein were significantly downregulated in A549 and H1299 cells treated with indicated concentrations of GK (*p* < 0.05; Western blotting). These results indicate that GK blocks the activation of AKT/GSK-3β/Snail in A549 and H1299 cells. Furthermore, In A549 and H1299 cells treated with the indicated concentrations of GK, the expression level of c-Myc protein was also significantly decreased (*p* < 0.05; Western blotting). These results indicate that GK can block Wnt/β-catenin activation in A549 and H1299 cells (Figure 10).

### 3.12 GK prevents tumor cells from spreading to the lungs in mice

**FIGURE 11:**
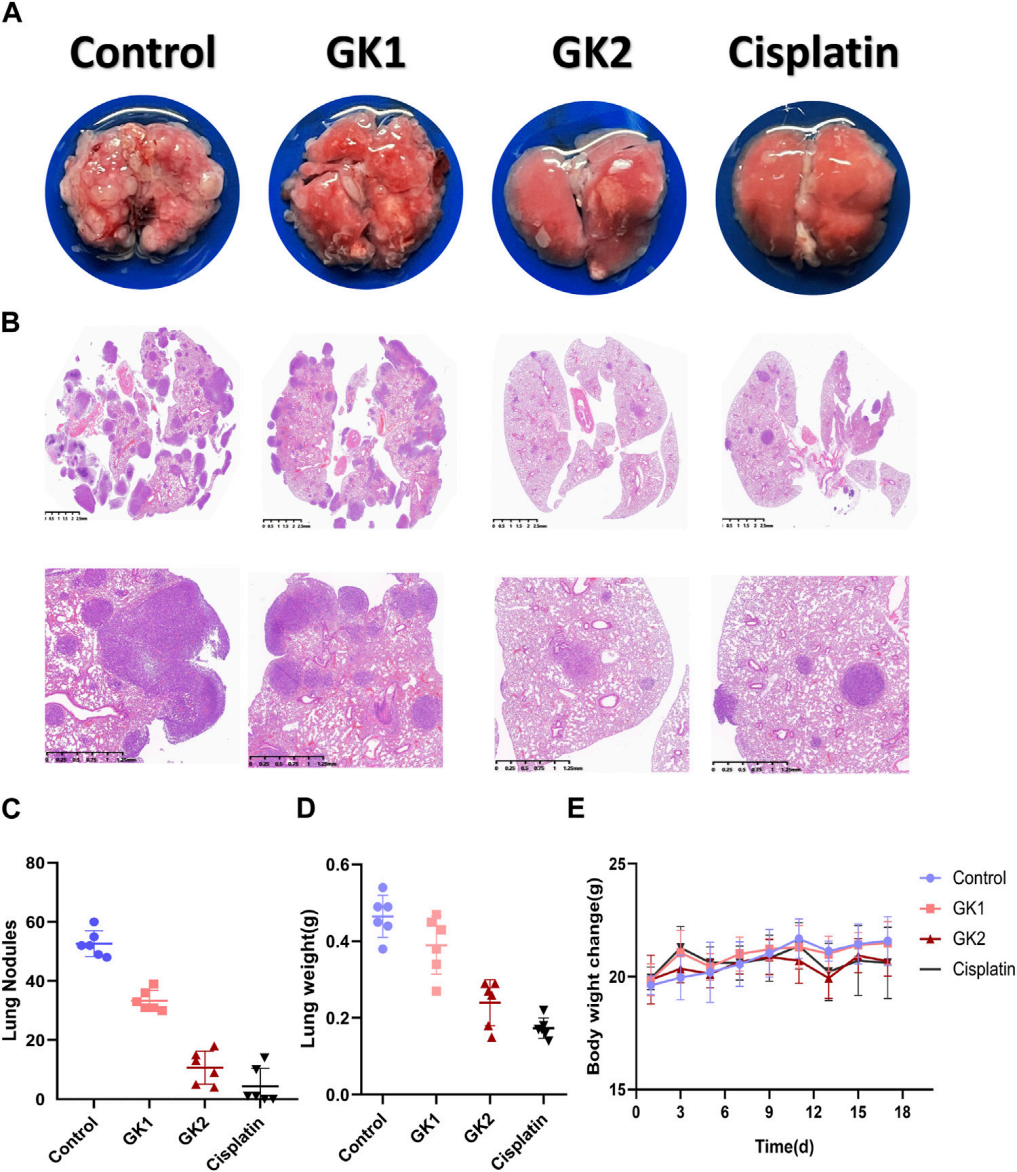
GK and Cisplatin suppresses the growth of lung metastases. **(A)**. 1.5×10^5^ LLC cells were injected into the tail vein of mice to establish a lung metastases model; *n* = 6 mice per group. The treatment started the day after the injection. Mice were sacrificed 14 days after injection. **(B)**. Representative lungs and HE staining (*n* = 6). **(C)**. Count of lung nodules in each group (*n* = 6). **(D)**. Weight of mouse lung. **(E)**. Change in body weight of mice.

To confirm the anti-metastatic efficacy of GK. LLC cells were injected into 7-week-old C57 mice *via* the tail vein and GK was injected into the mice. We found that the number of lung tumor nodules was lower in both the GK group than in the control group (Figure 11A). Meanwhile, HE staining showed that the lung tumor burden in the GK group was significantly lower than that in the control group (Figure 11B). These findings suggest that GK can effectively prevent tumor cells from spreading to the lungs (Figures 11C–E).

## 4 Discussion

One of the primary causes of mortality for people with lung cancer is metastasis, making it one of the deadliest cancers in the world (Zhao et al., 2021). However, the efficacy of the anticancer drugs on the current medical market is insignificant; therefore, it is essential to continue delving into anticancer treatment research and discover effective anticancer drugs and methods. While there are difficulties in studying the mechanisms of GK’s anticancer effects based on herbal pharmacology, network pharmacology based on big data and computer technology allows for comprehensive and rapid analysis of pharmacological mechanisms of action, addressing some of these obstacles (Luo et al., 2020). Network pharmacology takes a holistic view of biological network balance to understand drug–organism interactions and guide new drug discoveries. The new paradigm of molecular drug design based on network pharmacology offers novel insights into the study of Chinese medicine’s complex systems. Its holistic and methodical character is consistent with the holistic perspective of TCM and the principles of diagnosis and treatment. In addition, its research philosophy fits with TCM’s holistic perspective, which is extensively employed in TCM research (Zhang et al., 2022). Many reports proved that adopting TCM databases combined with computer software has improved TCM’s innovation and development levels and enhanced its international status. Through enrichment analysis, we identified the pathway (hsa05200) and the proteoglycan pathway in cancer (hsa05205) as the main pathways through which the active GK components exert their anticancer effects. The GO enrichment study’s findings suggested that the active GK components may exhibit anticancer effects by favorably regulating cell migration.

We built a protein interaction network of intersecting genes using the STRING database to clarify how GK inhibits lung cancer cell migration and invasion. PIK3R1, which controls cancer cell proliferation, is related to tumor growth and metastasis; PIK3R1 or PIK3R2 plays a role in the pathogenesis of distinct malignancies *via* common molecular processes. (Liu et al., 2022c). PI3K/AKT signaling system is substantially active in carcinogenesis; PIK3CA, PIK3R1, PTEN, AKT, and other genes have high-frequency mutations (PIK3CA gene is mutated in around 36% of breast tumors). These mutations are strongly related to tumorigenesis, progression, and medication resistance. (Chen et al., 2018). During cell migration, GSK-3β kinase activity can be regulated by upstream signals such as AKT (Buttrick and Wakefield, 2008). When GSK-3β (Ser9) is phosphorylated, Snail inhibition is released, increasing its expression level in cells (Liu et al., 2014b). The typical EMT markers are Snail and E-cadherin. Snail can decrease E-cadherin transcription and expression by binding to the E-cadherin promoter region, reducing intercellular adhesion (Liu et al., 2014a). AKT1 knockdown inhibits A549 cells’ survival, proliferation, malignancy, and metastasis (Jere et al., 2009). In our study, the expression level of β-catenin was downregulated. In the Wnt/β-catenin pathway, GSK-3β is an upstream molecule of β-catenin. GSK-3β can phosphorylate β-catenin and prevent β-catenin from entering the nucleus for transcription by ubiquitination and degradation. The above results suggest that GK enhances GSK-3β protein activity and inhibits the Wnt/β-catenin pathway. The downregulation of total GSK-3β and upregulation of phosphorylated GSK-3β suggest that there may be a negative feedback system to regulate GSK-3β protein, which is similar to a “braking system”. This may be a new discovery for us. However, we are currently unable to find out a molecule that regulates GSK-3β with negative feedback.

**FIGURE 12:**
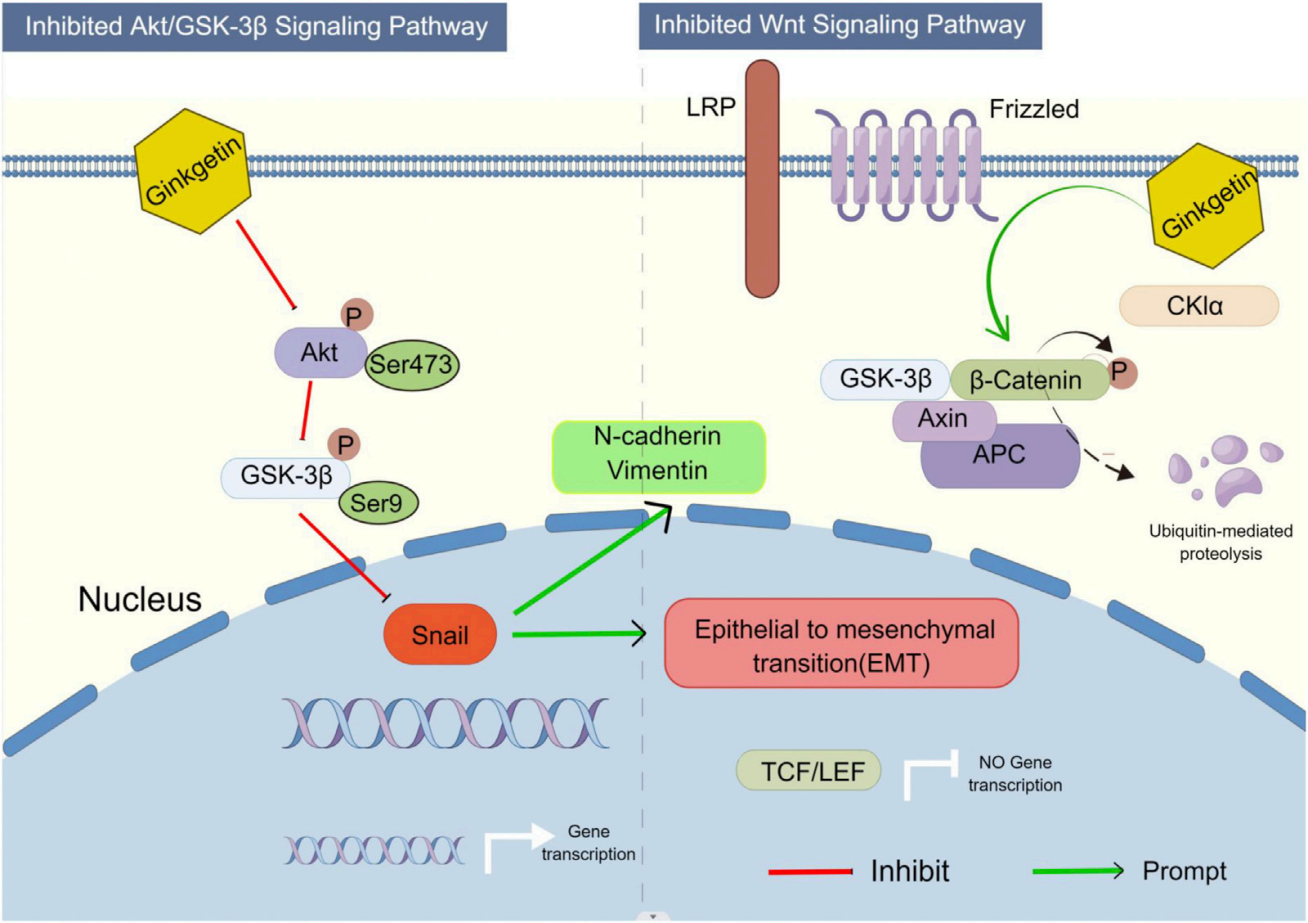
Proposed model illustrates how GK inhibits EMT in A549 and H1299 cells *via* the AKT/GSK-3β/Snail axis and Wnt/β-catenin axis (By Figdraw).

In cancer cell invasion and metastasis, epithelial cells undergo EMT, where they lose their epithelial properties and acquire a migratory behavior enabling them to move away from their epithelial cell population and integrate into surrounding tissues, even at distant sites. EMT is regarded as a crucial step in tumor progression. (Xu et al., 2009). Studies have shown that the AKT/GSK-3β/Snail signaling pathway is associated with EMT and metastasis in various malignancies. (Zhang et al., 2019). The current research findings reveal that GK may prevent the activation of the Akt/GSK-3β/Snail cascade in A549 cells, exerting an anti-invasive metastasis effect. Previous studies suggest that the AKT/GSK-3β/snail signaling pathway may trigger EMT (Xu et al., 2015; Zhou et al., 2015). GK inhibits the Akt/GSK-3β/Snail pathway and Wnt/β-catenin pathway to prevent lung cancer invasion and spread (Figure 12). The results of this study are consistent with the expectation of network pharmacology, which is widely used in TCM research, and provide a new concept for studying the complex system of TCM, and we found that there may be a “brake system” to regulate GSK-3β with negative feedback, which provides a new scientific support for rational clinical use and innovative drug development.

## 5 Conclusion

This study identified the potential related GK molecular targets and signaling pathways in treating human lung cancer using network pharmacological approaches. According to network pharmacological analysis, 79 genes/proteins were indicated to be possible GK targets. Moreover, a bioinformatic examination of projected targets found over 154 pathways. Experiments confirmed that GK inhibits the Akt/GSK-3β/Snail and Wnt/β-catenin cascade initiation in A549, H1299 and LLC cells, preventing metastasis. This study’s results align with the hypotheses derived from the network pharmacology analysis.

## Data Availability

The original contributions presented in the study are included in the article/supplementary material, further inquiries can be directed to the corresponding authors.

## Abbreviations

EMT, epithelial-to-mesenchymal transition; FBS, fetal bovine serum; GK, Ginkgetin; GO, gene ontology; KEGG, Kyoto Encyclopedia of Genes and Genomes; NSCLC, non-small cell lung cancer; SCLC, small cell lung cancer.
